# Neuronal Count, Brain Injury, and Sustained Cognitive Function in 5×FAD Alzheimer’s Disease Mice Fed DHA-Enriched Diets

**DOI:** 10.3390/biom15081164

**Published:** 2025-08-14

**Authors:** Cristina de Mello-Sampayo, Mafalda Soares Pádua, Maria Rosário Silva, Maria Lourenço, Rui M. A. Pinto, Sandra Carvalho, Jorge Correia, Cátia F. Martins, Romina Gomes, Ana Gomes-Bispo, Cláudia Afonso, Carlos Cardoso, Narcisa Bandarra, Paula A. Lopes

**Affiliations:** 1 iMED.UL, Faculdade de Farmácia, Universidade de Lisboa, Avenida Professor Gama Pinto, 1649-003 Lisboa, Portugal; csampayo@ff.ulisboa.pt (C.d.M.-S.); mariaqrs@gmail.com (M.L.); 2 Faculdade de Farmácia, Universidade de Lisboa, Avenida Professor Gama Pinto, 1649-003 Lisboa, Portugal; rapinto@ff.ulisboa.pt; 3 CIISA—Centro de Investigação Interdisciplinar em Sanidade Animal, Faculdade de Medicina Veterinária, Avenida da Universidade Técnica, 1300-477 Lisboa, Portugal; mafaldapadua@fmv.ulisboa.pt (M.S.P.); mrosariorsilva@gmail.com (M.R.S.); sandracarvalho@fmv.ulisboa.pt (S.C.); jcorreia@fmv.ulisboa.pt (J.C.); 4 AL4AnimalS—Laboratório Associado para Ciência Animal e Veterinária, Avenida da Universidade Técnica, 1300-477 Lisboa, Portugal; 5 JCS, Dr. Joaquim Chaves, Laboratório de Análises Clínicas, Rua Aníbal Bettencourt, Edifício Core, 3, 2700-225 Carnaxide, Portugal; 6 LEAF—Linking Landscape, Environment, Agriculture and Food Research Center, Instituto Superior de Agronomia, Universidade de Lisboa, Tapada da Ajuda, 1349-017 Lisboa, Portugal; catiamartins@isa.ulisboa.pt; 7 TERRA—Laboratório Associado, Instituto Superior de Agronomia, Universidade de Lisboa, Tapada da Ajuda, 1349-017 Lisboa, Portugal; 8 Divisão de Aquacultura, Valorização e Bioprospeção (DivAV), Instituto Português do Mar e da Atmosfera (IPMA, I.P.), Avenida Alfredo Magalhães Ramalho, 6, 1495-165 Algés, Portugal; rominamgomes@hotmail.com (R.G.); ana.bispo@ipma.pt (A.G.-B.); cafonso@ipma.pt (C.A.); carlos.cardoso@ipma.pt (C.C.); narcisa@ipma.pt (N.B.); 9 MEtRICs/Departamento de Química, NOVA School of Science and Technology, FCTNOVA, Universidade Nova de Lisboa, Campus de Caparica, 2829-516 Caparica, Portugal; 10 CIIMAR—Centro Interdisciplinar de Investigação Marinha e Ambiental, Universidade do Porto, Rua dos Bragas 289, 4050-123 Porto, Portugal

**Keywords:** Alzheimer’s disease, 5×FAD transgenic mice, DHA-enriched diets, cognitive decline, neuronal count, brain immunohistochemistry, fatty acid profile

## Abstract

Alzheimer’s disease (AD) is the most common form of dementia, affecting over 50 million people globally. Since 1906, efforts to understand this neurodegenerative disease and to develop effective treatments have continued to this day. Recognizing docosahexaenoic acid (DHA, 22:6n-3) as a safe, inexpensive and vital nutrient for brain health and cognitive protection due to its key role in brain development and function, this study explores novel, sustainable non-fish sources as potential dietary supplements to prevent or mitigate AD, within a blue biotechnology framework. Forty 5×FAD male mice, five weeks old, were allocated to five body weight-matched dietary groups (*n* = 8) and fed isocaloric diets based on AIN-93M standard chow for 6 months. Each diet, except the control feed (non-supplemented group), enclosed a modified lipid fraction supplemented with 2% of the following: (1) linseed oil (LSO, rich in alpha-linolenic acid (ALA,18:3n-3)); (2) cod liver oil (fish oil, FO, rich in both DHA and eicosapentaenoic acid (EPA, 20:5n-3)); (3) *Schizochytrium* sp. microalga oil (Schizo) with 40% of DHA; and (4) commercial DHASCO oil (DHASCO) with 70% of DHA. The different diets did not affect (*p* > 0.05) growth performance criteria (e.g., final body weight, daily feed intake, and body weight gain) suggesting no effect on the overall caloric balance or mice growth, but n-3 long-chain polyunsaturated-fatty acid (n-3 LCPUFA) supplementation significantly reduced total cholesterol (*p* < 0.001) and total lipids (*p* < 0.001). No systemic inflammation was detected in 5×FAD mice. In parallel, a beneficial modulation of lipid metabolism by DHA-enriched diets was observed, with polyunsaturated fatty acid incorporation, particularly DHA, across key metabolic tissues, such as the liver (*p* < 0.001) and the brain (*p* < 0.001). No behavioural variations were detected using an open-field test after 6 months of diet (*p* > 0.05). While mice fed a standard diet or LSO diet showed cognitive deficit, the incorporation of FO, Schizo or DHASCO oils into dietary routine showed promising protective effects on the working memory (*p* < 0.05) and the last two diets also on the recognition memory (*p* < 0.05) Increased neuronal count (*p* < 0.05), reflecting neuronal survival, was clearly observed with the fish oil diet. In turn, the number of TAU-positive cells (*p* < 0.05) was reduced in the Schizo diet, while β-amyloid deposition (*p* < 0.01) and the neuroinflammatory marker, IBA1 (*p* < 0.05), were decreased across all DHA-enriched diets. These promising findings open new avenues for further studies focused on the protective effects of DHA derived from sustainable and underexploited *Schizochytrium* sp. microalga in the prevention of AD.

## 1. Introduction

Rising life expectancy and declining birth rates are contributing to a sharp increase in the median age of the European population, leading to major challenges concerning senior citizens’ health. Until 2050, the number of people aged over 60 years will double (https://www.who.int/health-topics/ageing#tab=tab_1, accessed on 5 May 2025). On the one hand, the risk of neurodegenerative diseases is known to rise sharply with age. On the other hand, neurocognitive development is strongly affected by dietary factors, including nutrition, as it plays a key role in maintaining brain health throughout the lifespan of an individual [1].

Docosahexaenoic acid (DHA, 22:6n-3), an essential n-3 long-chain polyunsaturated fatty acid (n-3 LCPUFA), is uniquely concentrated in the brain, and it is essential for normal neurological development, assuring optimal neuronal function [2,3,4]. The deficiency of DHA has adverse effects on cognition and synaptic plasticity [5] and has been strongly associated with neurological disorders, including Alzheimer’s disease (AD) [6,7].

AD is the most common cause of dementia worldwide outlined by gradual and progressive memory impairment, deterioration of other cognitive capacities, and inability to carry out daily routine activities [8,9,10]. AD is a neurological multifactorial disorder described by synaptic damage and neuronal loss, impacting mostly the cortex and hippocampus [11,12] with β-amyloid plaque deposition and neurofibrillary tangles, turning these features into the pathophysiological hallmarks of AD [11,13,14]. β-amyloid plaques are the result of this peptide oligomer accumulation, triggered by a disrupted cleavage of the amyloid precursor protein (APP) by α-, β-, and γ-secretases and/or reduced degradation of β-amyloid, which, because of their native misfolded nature, depict numerous chemical groups classified as toxic [8,12,14,15,16,17], whereas neurofibrillary tangles are hyperphosphorylated variants of the microtubule-associated tau protein deriving from the imbalanced action of phosphatase enzymes and protein kinases, which accumulate in the neuron’s body and dendrites, completely disrupting its structure and compromising its functionality [8,12,14,18,19]. Regardless of being considered a major worldwide public health concern and the rise in exceptional advances during the last decades for Alzheimer’s disease understanding, no effective treatment exists to cure AD patients [20,21,22].

In line with this, international nutritional guidelines have recommended increasing DHA intake. Oily fish has been considered for decades the best source of n-3 LCPUFA [2]. However, current n-3 LCPUFA sources are unsustainable because the existing supplies of wild fish are insufficient to meet the recommended intake values [23] pushing their sustainability to the limit, not to mention other existent important limitations, such as dietary and cultural habits, the concern about the presence of contaminants, including mercury in some fish species, and the high cost versus low oxidative stability of fish oils included in food matrices, which eventually affect the organoleptic and nutritional properties of the final food product [24,25]. Facing this discussion, it is imperative to look for alternative sources of n-3 LCPUFA to increase DHA bioavailability. Microalgae could be the answer. Microalgae stand out as a viable, sustainable, and eco-friendly source of n-3 LCPUFA, containing their oils up to 40% of a single FA (% total FA) [26]. New improvements in large-scale production of microalgae are presently under development to efficiently produce highly pure DHA oils [27], as the case of thraustochytrid cells typically characterized by a high proportion of DHA, without eicosapentaenoic acid (EPA, 20:5n-3), in the total FA [27].

Assuming the premise that n-3 LCPUFAs as DHA are a safe and inexpensive link to a healthier long life, this study aims to explore the potential of novel and sustainable non-fish sources from blue biotechnology for human dietary supplementation [28,29]. The elucidation of individual biological effects of DHA from microalga oils, such as *Schizochytrium*-microalga oil and commercial DHASCO oil, by targeting the brain across key metabolic pathways of disease, on the prevention and treatment of neurodegenerative pathologies, such as AD, is a major topic underscoring the relevance of key nutrients in supporting neuroprotection. The production of DHA pure oils from microalgae will clarify whether DHA has individual health benefits or if such benefits are cumulative or interactive with EPA, since their presence in fish is always mutual, thus providing sustained information to guide their individual or combined consumption. In fact, apart from a few reports [30,31,32], the vast majority of studies assessing n-3 LCPUFA biological effects are based on fish products and, therefore, on the effects of EPA and DHA in combination. This remains to be elucidated.

In this study, we hypothesized that DHA-enriched diets derived from *Schizochytrium* sp. microalga can overcome the proven benefits of fish oil intake across neuronal count, brain injury, and cognitive decline in a traditional experimental mouse model for AD research.

## 2. Materials and Methods

### 2.1. Ethics Statement

This investigation was conducted in compliance with the European Union (Directive 2010/63/EU) regulatory framework. It received consent from the Ethics Review Board of CIISA/FMV, the Animal Welfare Committee of the National Veterinary Authority (Direção Geral de Alimentação e Veterinária (DGAV, Portugal, ref.: 20163/23-S approved on 2 November 2023), and ORBEA/CIISA (ref.: 001/2023 approved on 19 May 2023). The ARRIVE guidelines 2.0 were followed in mice experimental trial (https://arriveguidelines.org/arrive-guidelines).

### 2.2. Mice, Study Design, and Experimental Diets

Forty 5×FAD male mice, five weeks old, were purchased from Charles River Laboratories (L’Arbresle, France). The mouse strain used for this research project, B6SJL-Tg(APPSwFlLon,PSEN1M146LL286V)6799Vas/Mmjax, RRID, was obtained from the Mutant Mouse Resource and Research Center (MMRRC) at The Jackson Laboratory, an NIH-funded strain repository. This strain was donated to the MMRRC by Robert Vassar, Ph.D., Northwestern University.

Upon arrival, mice were single-housed in the animal facility of the Faculty of Veterinary Medicine, University of Lisbon, under controlled environmental conditions, such as room temperature of 22 ± 2 °C, relative humidity of 55 ± 5%, and a 12/12 h light–dark cycle (lights on at 8:00 a.m.) [33]. After the first week of acclimation, under a standard maintenance purified diet (AIN-93M, Envigo, Spain) to minimize stress and stabilize all metabolic conditions, mice were assigned to five body weight-matched dietary groups with 8 animals each and fed isocaloric diets based on AIN-93M standard chow for rodents for 6 months. Each diet, with the exception of the control feed (non-supplemented group, control), encloses a modified lipid fraction supplemented with 2% of the following: (1) linseed oil (LSO, rich in alpha-linolenic acid, the precursor of omega-3 LCPUFA pathway, taken as the negative control); (2) cod liver oil (fish oil, FO, rich in both DHA and EPA, taken as the positive control); (3) *Schizochytrium* sp. microalga oil (Schizo) with 40% of DHA oil; (4) and commercial DHASCO oil (DHASCO) with 70% of DHA oil also derived from *Schizochytrium* sp. microalga. The oils were purchased from the following: linseed (flax) oil, ref.: 0140000, De Wit Speciality Oils, the Netherlands; cod liver oil, ref.: CNP 7749945, Labsolve, José Manuel Gomes dos Santos, Lisbon, Portugal; NEWmegaTM Algae Oil, ref.: 0603640, De Wit Speciality Oils, the Netherlands; and DHASCO oil, ref.: 6217-54-5, Dideu Industries, China. The experimental diets were manufactured by Sparos company (Olhão, Portugal).

Each diet contained the following components (g/100 g of dry matter): corn starch (46.6), maltodextrin (15.5), casein (14.0), sucrose (10.0), cellulose (5.0), soybean oil (4.0), AIN-93M mineral mix (3.5), AIN-93M vitamin mix (1.0), choline bitartrate (0.25), L-cystine (0.18), and tert-butylhydroquinone (0.0008). The proximate chemical composition of the diets was determined according to the Association of Official Analytical Chemists [34], and the fatty acid composition was assessed as described by the authors of [35] (Table 1).

Diet samples were stored frozen at −20 °C and protected from light until analysis. The granules from the diet samples were first cut into small pieces and then ground using a mill (Ultra Centrifugal Mill ZM 200, Retsh, Haan, Germany) with a sieve diameter of 1 mm. Dry matter was determined by drying the samples in an oven at 103 °C to constant weight. Ash was determined by burning the samples in a muffle furnace at 525 °C. Crude protein was determined according to the Kjeldahl method by multiplying the nitrogen content by a factor of 6.25. Crude fat was determined by automated Soxhlet extraction with petroleum ether (Tecator Soxtec System extraction unit, Tecator AB, Sweden), after acid hydrolysis pre-treatment in hydrolysing unit (Tecator Soxtec, Tecator AB, Sweden). Crude fibre was obtained after the acid and basic hydrolysis of the samples using a hot extraction Fibertec (model FT 122, Foss A/S, Hillerød, Denmark). The above determinations were carried out in accordance with AOAC [34] guidelines, specifically methods 924.01, 942.05, 954.01, 954.02, and 978.10 for dry matter, ash, crude protein, ether extract and crude fibre, respectively. Gross energy content was determined by adiabatic bomb calorimetry (Parr 6400, Parr Instrument Company, Moline, IL, USA), as previously described by the authors [36]. All diets were analyzed in triplicate, and results are expressed as the mean ± standard deviation. Interventionary studies involving animals or humans, as well as other studies that require ethical approval, must list the authority that provided approval and the corresponding ethical approval code.

**Table 1:** Chemical composition, energy content, and fatty acid composition of n-3 PUFA-enriched diets.

	Control	LSO	FO	Schizo	DHASCO
Gross energy (kcal/kg)	4001 ± 1.32	4067 ± 24.6	4041 ± 9.47	4076 ± 2.65	4066 ± 4.00
Proximate composition (g/100 g)					
Dry matter	91.6 ± 0.040	90.8 ± 0.085	90.8 ± 0.012	91.2 ± 0.081	91.0 ± 0.012
Crude protein	13.1 ± 0.112	12.9 ± 0.418	12.2 ± 0.127	12.6 ± 0.135	12.4 ± 0.112
Crude fat	4.23 ± 0.031	5.60 ± 0.060	5.55 ± 0.054	5.48 ± 0.119	5.74 ± 0.057
Carbohydrates *	71.7 ± 0.124	69.9 ± 0.432	70.6 ± 0.139	70.7 ± 0.199	70.5 ± 0.128
Crude fibre	3.43 ± 0.053	3.23 ± 0.038	3.09 ± 0.143	3.23 ± 0.161	3.13 ± 0.037
Ash	2.53 ± 0.011	2.42 ± 0.022	2.43 ± 0.011	2.45 ± 0.024	2.42 ± 0.021
Total lipids (%)	3.99 ± 0.439	5.96 ± 0.232	6.38 ± 0.062	4.26 ± 0.799	5.36 ± 0.062
Lipid classes (%)					
PL	2.74 ± 0.71	1.17 ± 0.09	1.67 ± 0.13	2.07 ± 0.31	0.00 ± 0.00
MAG	0	0	0	0	0
1,2 DAG	4.42 ± 0.28	4.90 ± 0.25	5.05 ± 0.41	4.10 ± 0.02	5.14 ± 0.18
1,3 DAG + CHR	12.9 ± 0.22	14.3 ± 0.51	10.2 ± 0.09	12.1 ± 0.12	13.2 ± 0.43
FFA	11.4 ± 0.41	9.58 ± 0.85	9.23 ± 0.97	9.84 ± 0.44	9.11 ± 0.16
TAG	68.5 ± 1.20	70.0 ± 0.76	73.8 ± 1.02	71.9 ± 0.63	72.6 ± 0.42
Fatty acid composition (% total fatty acids)					
12:0	0.02 ± 0.04	0.01 ± 0.02	0.05 ± 0.00	0.16 ± 0.01	0.03 ± 0.03
13:0	0.18 ± 0.15	0.16 ± 0.02	0.20 ± 0.02	0.18 ± 0.03	0.18 ± 0.00
14:0	0.25 ± 0.02	0.18 ± 0.01	1.17 ± 0.03	2.04 ± 0.15	0.25 ± 0.02
16:0	12.39 ± 1.39	9.71 ± 0.01	11.39 ± 0.05	12.87 ± 0.18	9.62 ± 0.10
16:1n-7	0.09 ± 0.08	0.10 ± 0.01	2.07 ± 0.08	1.88 ± 0.13	0.08 ± 0.07
17:0	0.07 ± 0.06	0.09 ± 0.00	0.10 ± 0.00	0.11 ± 0.00	0.05 ± 0.05
18:0	4.74 ± 0.22	4.70 ± 0.13	3.94 ± 0.04	3.84 ± 0.15	3.81 ± 0.02
18:1n-9	22.72 ± 0.38	21.92 ± 0.41	21.20 ± 0.10	18.74 ± 0.43	18.47 ± 0.11
18:1n-7	1.36 ± 0.04	1.15 ± 0.01	2.12 ± 0.03	2.63 ± 0.06	1.11 ± 0.01
18:2 n-6	50.67 ± 0.44	40.28 ± 0.10	38.12 ± 0.37	38.93 ± 0.59	40.51 ± 0.41
18:3 n-3	5.76 ± 0.18	20.07 ± 0.52	4.60 ± 0.05	4.46 ± 0.08	4.65 ± 0.06
18:4 n-3	nd	0.03 ± 0.05	0.54 ± 0.02	0.10 ± 0.03	nd
20:0	nd	0.30 ± 0.02	0.26 ± 0.01	0.30 ± 0.02	0.33 ± 0.00
20:1 n-9	0.17 ± 0.01	0.15 ± 0.01	2.92 ± 0.08	0.19 ± 0.02	0.15 ± 0.02
20:4 n-6	nd	nd	0.12 ± 0.01	0.04 ± 0.04	0.18 ± 0.00
20:5 n-3	nd	nd	2.59 ± 0.09	0.42 ± 0.02	0.12 ± 0.10
22:0	0.35 ± 0.02	0.29 ± 0.01	0.28 ± 0.00	0.30 ± 0.04	0.31 ± 0.01
22:5 n-6	nd	nd	nd	1.67 ± 0.05	3.37 ± 0.07
22:6 n-3	nd	nd	2.90 ± 0.09	10.01 ± 0.40	15.86 ± 0.37
SFA	18.38 ± 1.44	15.44 ± 0.16	17.64 ± 0.03	19.98 ± 0.23	14.60 ± 0.14
MUFA	24.37 ± 0.43	23.35 ± 0.39	31.63 ± 0.16	23.52 ± 0.33	19.84 ± 0.11
PUFA	56.50 ± 0.51	60.43 ± 0.52	49.84 ± 0.20	55.75 ± 0.27	64.80 ± 0.15
n-3 PUFA	5.76 ± 0.18	20.09 ± 0.54	11.24 ± 0.17	15.05 ± 0.43	20.71 ± 0.49
n-6 PUFA	50.67 ± 0.44	40.30 ± 0.08	38.33 ± 0.36	40.65 ± 0.52	44.06 ± 0.35
n-3/n-6	0.11 ± 0.00	0.50 ± 0.01	0.29 ± 0.01	0.37 ± 0.01	0.47 ± 0.01

* Carbohydrates were determined by difference, as described by FAO [37]. Control: AIN-93M diet; LSO: linseed oil diet; FO: cod liver oil diet; Schizo: *Schizochytrium* microalga oil diet; DHASCO: commercial DHASCO oil diet; PL: phospholipid; MAG: monoacylglycerol; 1,2 DAG: 1,2 diacylglycerol; 1,3 DAG + CHR: 1,3 diacylglycerol plus cholesterol; FFA: free fatty acid; and TAG: triacylglycerol. nd: not detected; SFA: saturated fatty acid; MUFA: monounsaturated fatty acid; PUFA: polyunsaturated fatty acid. Results are expressed as the mean ± standard deviation of 3 replicates.

Throughout the experimental trial, mice had free access to water and food. Body weight and food intake were recorded weekly. After 6 months of feeding trial, mice were fasted for 12 h and then euthanized by a mechanical–physical method. Mice were placed in a chamber and anesthetized using a mixture of 20% isoflurane in propylene glycol (*v*/*v*) for 30 s, followed by decapitation with a small animal guillotine. The trunk blood was collected into lithium heparin tubes (Sarstedt, Nümbrecht, Germany) and centrifuged (during 10 min at 1500× *g*, RT) to isolate plasma. Following blood collection, half of the brain was excised and fixed by immersion in 10% neutral buffered formalin (Merck, VWR International, PA, USA) for 24 h and then processed for paraffin embedding (Microscopy Histosec, Merck). The other half of the brain was collected for fatty acid profiling and kept at −80 °C until analysis.

### 2.3. Behavioural Testing

Behavioural tests included the T-Maze, the open-field test (OF), and the novel object recognition test (NOR). The T-Maze test was performed at both 1 and 6 months into the feeding trial, whereas the NOR and the OF tests were conducted exclusively at 6 months of nutritional intervention, when the animals were 7.5 months old.

Regarding this aspect, the review manuscript recently published by Pádua et al. [22] reports insightful information on 5×FAD mouse model, which recapitulates AD development, with high levels of β-amyloid and intraneuronal deposition by the age of 1.5 months until the appearance of substantial cognitive deficits by the age of 4 months. However, this timeline is not consensual as other authors reported the existence of behavioural impairment between 4 and 6 months old [38]. With this in mind, and considering that the nutritional intervention herein proposed might be more protective in AD earlier stages instead of AD advanced stages, the authors set 6 months old as the age at which it would be the best time, under these experimental nutritional settings, to carry out behavioural assessment.

Prior to testing, mice were familiarized to handlers and adapted to the testing room once a day during 2–3 successive days. To acclimatize them to the testing environment, mice were placed into the testing room at least 1 h before testing. All apparatuses were cleaned with 30% ethanol between animals and sessions. Researchers conducting the behavioural testing were blinded to the dietary groups to ensure unbiased evaluation.

#### 2.3.1. T-Maze Test

T-maze test was conducted in a room dimly lit with an intensity of 25 lx in each maze arm and following a spontaneous alternation protocol identical to the one reported by d’Isa et al. [39]. Briefly, animals were placed in the starting arm facing away from the goal arms. The timer started when the mice turned to the centre of the maze, and the time taken to choose an arm was recorded when all paws and tail were inside one of the goal arms. Mice were confined in the chosen arm for 30 s for adaptation. This process was repeated for trials 1–6 with an inter-trial interval of 35 s. Successful alternation was defined as choosing the arm not visited in the previous round, and alternation rate was computed using the formula: (number of correct alternations/5) × 100. Alternation rate offered an index for short-term memory. The choice latency and the defecation index (defined as the number of fecal boli/time) were measured to exclude a potential impact of motor impairment or emotional instability on the results.

#### 2.3.2. Open-Field Test

Baseline motor activity and exploratory and anxiety-like behaviour were assessed before the NOR test using the open-field (OF) test. Mice were placed individually in a closed arena (~30 cm × 30 cm × 30 cm width, length, and height), and the exploratory behaviour of mice was video-recorded for 5 min with a camera placed at the top of the apparatus. No prior training sessions were conducted before the test. An automated system (ANY-maze software, version 7.50, Stoelting Co., Wood Dale, IL, USA) measured the distance travelled (m) and time (s) spent in the centre and periphery of the field. The behavioural parameters of mice were also registered, including rearing, grooming, and jumping. The room was dimly lit with an apparatus intensity of 20 lx.

#### 2.3.3. Novel Object Recognition Test

The recognition memory of mice was evaluated using the novel object recognition (NOR) Test, following the protocol published by Bevins and Besheer [40]. This test was conducted in the same apparatus used for the OF test under the same lighting settings and included two phases separated by a 24 h interval. On the 1st day, during the familiarization phase, mice were exposed for 3 min to two identical objects (F and F′) placed in the back corners of the arena. On the 2nd day, during the test phase, which lasted 3 min, mice were placed in the arena containing one novel (N) object and the previously encountered familiar (F) object (N and F). Mice returned to their home cages between phases. The exploratory movement during both phases was recorded with a video camera installed at the top of the apparatus.

The variables measured in this behavioural test included the frequency of rearing and grooming, as well as the exploration time and frequency of both familiar (F) and novel (N) objects. The object exploration was defined as the nose of the mouse either touching or being directed to the object at a distance of less than 1 cm. Climbing or sitting on the top of an object was not recorded as exploration unless the nose was directed towards it. Mice showing too much freezing behaviour, corresponding to less than 10 s of interaction, were excluded from the analysis (1 mouse per group). Lack of side bias was also guaranteed, with no mice showing a side preference. For memory and cognitive analysis, the total time of object interaction was recorded, and the discrimination index was evaluated by the time spent exploring the novel object relative to the familiar object, calculated as the difference in time spent exploring the novel (TN) and familiar (TF) objects.

### 2.4. Plasma Biochemistry Profile

Plasma metabolites, such as glucose, insulin, urea, creatinine, total protein, triacylglycerols (TAG), total cholesterol, HDL cholesterol, and LDL cholesterol, as well as the activities of alanine aminotransferase (ALT, EC 2.6.1.2), aspartate aminotransferase (AST, EC 2.6.1.1), and gamma-glutamyltransferase (GGT, EC 2.3.2.13) were quantified with diagnostic kits from Roche Diagnostics’ Modular Hitachi Analytical System (Mannheim, Germany). The original formula by Friedewald et al. [41] was used to calculate VLDL cholesterol, and the derived formula by Covaci et al. [42] was used to calculate total lipids. The C-reactive protein was determined by immunoturbidimetry (Roche Diagnostics, Meylan, France). Interleukin-6 (IL-6) levels were measured using an electrochemiluminescence immunoassay (ECLIA) with a lower detection limit of 1.5 ng/L. Insulin-like growth factor-1 (IGF-1) was quantified using a chemiluminescence immunoassay (CLIA) with a sensitivity below 7.00 µg/L.

### 2.5. Histology and Immunohistochemistry in the Brain

Serial tissue sections (3 μm thick) from each of the paraffin-embedded specimens were cut with a Minot microtome (Leica RM2135, Nussloch, Germany). The sections were stained with classical Harris hematoxylin (Bio-Optica, Milan, Italy) and eosin-floxin (Bio-Optica) to assess morphology under a light microscope (Olympus BX51 equipped with Olympus DP21 microscope digital camera system, Olympus, Tokyo, Japan). Neurons were counted in 20 fields per section at a magnification of ×400 using ImageJ, software tools, version 1.54p.

For immunohistochemistry assays, the brain sections were subjected to antigen retrieval with citrate buffer (pH 6.0) at 96 °C for 20 min using PTLink equipment (DAKO, LusoPalex, Portugal). The sections were washed 2× with distilled water for 5 min, treated with H_2_O_2_ to quench endogenous peroxidase activity (Envision FLEX Kit, catalogue #K8000, DAKO, LusoPalex, Portugal) for 15 min, and then washed 2× in PBS for 5 min. The sections were incubated with β-amyloid recombinant rabbit monoclonal antibody (H31L21) (1:500, Invitrogen, ThermoScientific, Waltham, MA, USA, catalogue #700254, RRID: AB_2532306), TAU recombinant rabbit monoclonal antibody (1:100, Invitrogen, ThermoScientific, catalogue #MA5-41098, RRID: AB_2898852), and IBA1 polyclonal antibody (1:100, Invitrogen, ThermoScientific, catalogue #PA5-27436, RRID: AB_2544912) for 1 h. After incubation, the sections were washed again 2× in PBS for 5 min, incubated with donkey biotinylated anti-rabbit IgG (H + L) cross-absorbed secondary antibody (1:500, Invitrogen, ThermoScientific, catalogue #31458, RRID: AB_228213) at RT for 30 min, washed 2× in PBS for 5 min, and developed with DAB (1:20, Envision FLEX Kit, DAKO, catalogue #K8000, LusoPalex, Portugal) for 5 min. Sections were washed 2× with distilled water for 5 min, stained with Harris hematoxylin (Bio-Optica) for 1 min, washed 2× with distilled water for 5 min, dehydrated with increasing concentrations of ethanol, cleared with xylene, and covered with Entellan mounting reagent (Merck, VWR International, Radnor, PA, USA).

Immunohistochemistry analysis using antibodies against Aβ, TAU, and IBA1 allowed for the quantification of β-amyloid plaque count and area, TAU count, and IBA1 count and area in 20 fields per section at a magnification of ×400 using the ImageJ software tools. The entire histologic protocol was adapted from Christensen and Pike [43].

### 2.6. Total Lipids and Lipid Classes in the Experimental Diets

Total lipid content in the diets was estimated gravimetrically, as described by Bligh and Dyer [44], while the lipid class distribution was determined by high-performance thin-layer chromatography (HPTLC), according to the protocol described by Gomes et al. [45]. Briefly, 4 μL of the lipid extracts (10 mg/mL) were applied in a Silica gel 60 F_254_ HPTLC plate (200 mm × 100 mm, 0.5 mm; Merck, Darmstadt, Germany) using Linomat 5 (CAMAG, Muttenz, Switzerland). The lipid classes were then separated using a mixture of n-hexane, diethyl ether, and acetic acid (65:35:1, *v*/*v*/*v*) and derivatized by immersion in a 5% (*w*/*v*) phosphomolybdic acid ethanolic solution. After staining, plates were heated at 100 °C for 1 h, and the bands quantified by densitometry with the TLC Scanner 4 (CAMAG, Muttenz, Switzerland) at 650 nm.

### 2.7. Fatty Acid Composition in the Liver, Brain, and Experimental Diets

Total lipids were extracted according to Folch et al. [46]. Fatty acid methyl esters (FAMEs) were obtained following Bandarra et al.’s [35] protocol by adding 5 mL of 5% acetyl chloride-methanolic solution. Tubes were kept in a water bath at 80 °C for 1 h. After tubes were cooled, 1 mL of Milli-Q water and 2 mL of n-heptane were added to the tubes; then, tubes were agitated using the vortex, and the layers were separated by centrifugation (at 3000× *g* for 3 min). The organic phase was isolated and filtered through anhydrous sodium sulphate. The final extract was analyzed by gas chromatography (GC, Scion 456-GC gas chromatograph, West Lothian, UK) equipped with a 30 m × 0.25 mm i.d, film thickness 0.25 μm, DB-WAX capillary column (Agilent Technologies, Santa Clara, CA, USA) with helium as the carrier gas, starting at 180 °C for 5 min, increasing to 220 °C at 4 °C/min, and holding for 24 min. The identification of fatty acids was performed based on their retention times using a standard mix (PUFA-3, Menhaden oil, Sigma-Aldrich, St. Louis, MO, USA) as internal reference.

### 2.8. Statistical Analysis

The statistical analysis was carried out with SAS [47] software, version 9.1 using the generalized linear mixed (GLM) model. The normality of data was checked by Kolmogorov–Smirnov test, and the variance homogeneity was verified by Levene’s test. Significant multiple comparisons were performed using the PDIFF option adjusted with the Tukey–Kramer test to find statistical differences among diets. Pearson’s correlation coefficients were calculated using the Proc CORR procedure of SAS to find linear relationships among parameters. The principal component (PC) analysis was performed, using the proc PRINCOMP of SAS, to assess relationships between variables. After data normalization, the analysis was based on the correlation matrix, and PC were considered significant if they contributed more than 5% of the total variance. Data are presented as mean and SEM (standard error of the mean). *p* < 0.05 was chosen as the cut-off for statistical significance between dietary groups.

Behavioural testing results were processed by one-way ANOVA with Tukey’s post hoc to compare behaviours within each dietary group. In addition, the alternation scores in the T-Maze test and discrimination indices (DIs) in the NOR test were analyzed using one-sample *t*-tests, comparing each group’s alternation score or DI to the chance level of 50% or chance exploration level of 0, correspondingly. The object exploration time was studied by one-way ANOVA corrected for multiple comparison with Sidak’s post hoc, or by paired *t*-tests, to compare the object exploration within each dietary group.

## 3. Results

### 3.1. Mice Body Weight and Feed Intake

Initial body weight of mice presented no significant differences among dietary groups (control = 23.5 g, LSO = 23.4 g, FO = 23.8 g, Schizo = 23.7, DHASCO = 23.5, SEM = 0.759 g, *p* = 0.998). By the end of the experimental assay, no significant effects of the diet were observed on mice final body weight (control = 32.5 g, LSO = 32.2 g, FO = 36.0 g, Schizo = 33.1 g, DHASCO = 32.1 g, SEM = 2.21 g, *p* = 0.709), daily feed intake (control = 3.70 g, LSO = 3.79 g, FO = 3.81 g, Schizo = 3.76 g, DHASCO = 3.75 g, SEM = 0.124 g, *p* = 0.978), or body weight gain (control = 8.99 g, LSO = 8.81 g, FO = 12.3 g, Schizo = 9.40 g, DHASCO = 8.63 g, SEM = 1.76 g, *p* = 0.578).

### 3.2. Behavioural Assessment

**Figure 1:**
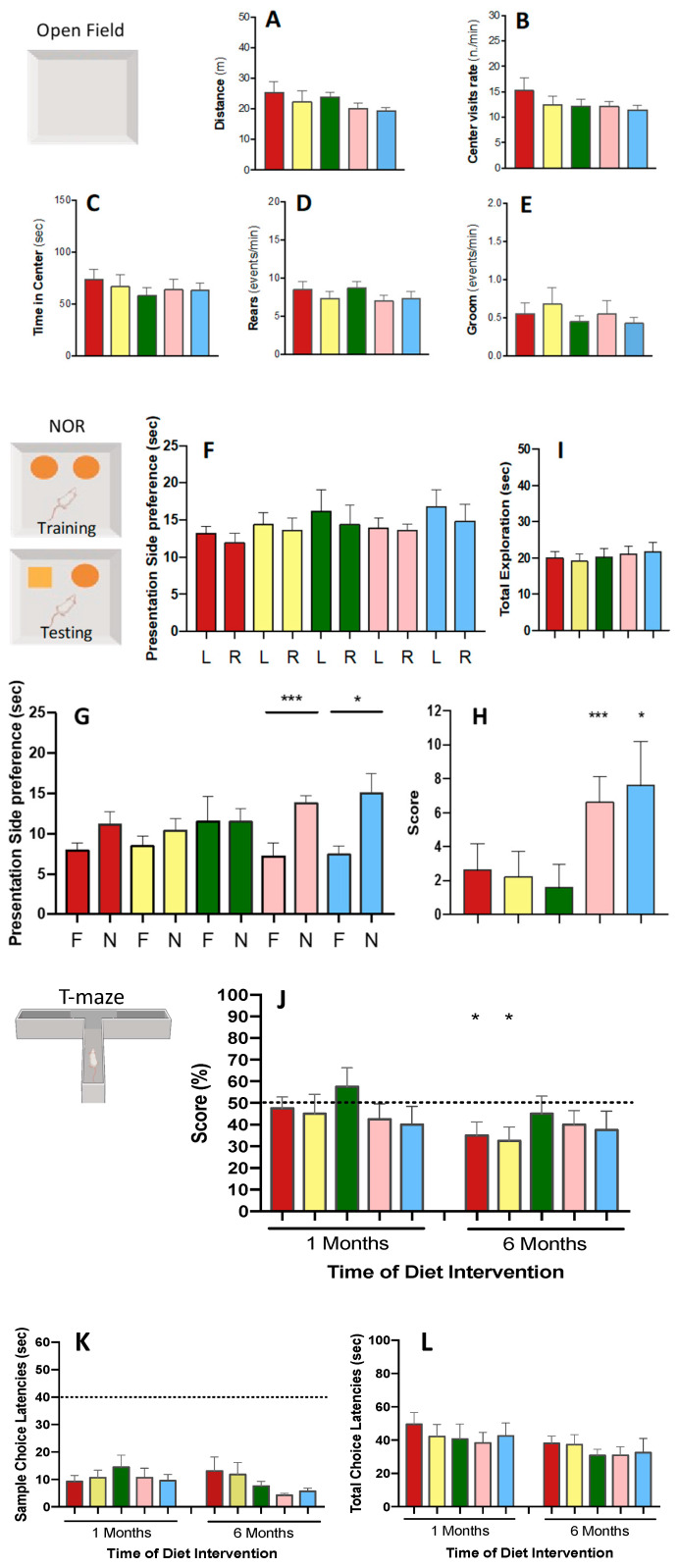
Dietary DHA-enriched diets sustained cognitive function in 5×FAD mice fed for 6 months. Standard (red), linseed oil (LSO, yellow), cod liver oil (FO, green), *Schizochytrium* microalga oil (pink), and DHASCO oil (blue). Data are mean ± SEM. Open-field test (**A**–**E**): (**A**) distance travelled; (**B**) centre visits; (**C**) time in centre; (**D**) rate of rear events; (**E**) rate of groom events. Novel object recognition (NOR) test (**F**–**I**): On the left side is shown the scheme of the arena and the position of objects. The circles mean familiar object. The square means novel object. (**F**) Exploration times for each object (L—left and R—right) during the familiarization phase. (**G**) Exploration times for each object (F—familiar and N—novel) during the test phase. (**H**) Discrimination score (TN-TF) (significantly above zero * *p* < 0.05 and *** *p* < 0.001). (**I**) Total exploration time. Spatial working memory in spontaneous alternation T-maze test (**J**–**L**) at 1 month and 6 months of dietary intervention: (**J**) spontaneous alternation (score) (* *p* < 0.05 compared with 50%); (**K**) choice latency; (**L**) total choice latencies; the dashed line represents the 50% chance level in (**J**) and the cut-off latencies in (**K**).

The effects of the experimental diets on spontaneous exploratory and memory-associated behaviour in 5×FAD mice are displayed in Figure 1. The open-field (OF) test assessed the baseline motor/exploratory activity, while memory-associated behaviour was evaluated through the novel object recognition (NOR) and T-maze tests.

#### 3.2.1. Open-Field Test

In the open-field (OF) test, which evaluated motor activity associated with anxiety-like behaviours, no significant differences were observed between dietary groups in horizontal activity (distance travelled: *p* = 0.4297; centre visit rate: *p* = 0.535) or vertical exploratory activity (rear rate: *p* = 0. 579), nor in emotional-like behaviour (time in centre: *p* = 0.838; grooming: *p* = 0.773) (Figure 1A–E). All in all, anxiety and stress were not experienced by 5×FAD mice, depending on the oil diets.

#### 3.2.2. Novel Object Recognition Test

The novel object recognition (NOR) was used to evaluate the recognition memory. During the adaptation phase, no differences were found in the exploration of identical objects or side preference (*p* = 0.839) (Figure 1F). On the other hand, in the test phase (Figure 1G), animals from all dietary groups—except the FO group—generally showed a preference for the novel object (‘N’) over the familiar (‘F’) one (*p* = 0.021). However, this increase in exploration time for the ‘N’ object was statistically significant only in Schizo and DHASCO dietary groups (*p* = 0.005 and *p* = 0.025, respectively). The discrimination index (DI) indicated a trend towards improved recognition memory in these groups, with the Schizo and DHASCO group presenting DI values 2.5 to 2.9 times higher than controls (*p* = 0.073) (Figure 1H). Notably, mice that were fed LSO or FO diets did not discriminate above zero, confirming a recognition memory deficit, while the Schizo and the DHASCO diet groups demonstrated a clear preference for the novel object (*p* = 0.001 and *p* = 0.012, respectively). Test exploration time (Figure 1I) was very similar across dietary groups (*p* = 0.474) as well as spontaneous behaviours, like rearing and grooming, during both familiarization (*p* = 0.345, *p* = 0.578) and test trials (*p* = 0.739, *p* = 0.484). These findings indicate that the observed exploratory behaviour was not due to mobility impairments or a lack of motivation. Overall, these discrimination results suggest that 5×FAD mice that were fed Schizo or DHASCO diets for 6 months retained memory for the novel object, unlike those on either standard diet or even LSO or FO oil diets.

#### 3.2.3. T-Maze Test

The T-maze test was used to study the spatial working memory by assessing the animal’s tendency to explore a new environment (spontaneous alternation). The test places the mouse at the end of an open arm of the maze, and both the latency to enter one of the other two arms and the alternation between arms are registered. For alternation scores, which indicate working memory, while near 60% correct choices were made by FO-fed mice, only about 50% were made by all other the 5×FAD mice following 1 month of intervention, regardless of diet treatment (Figure 1J). Between the treated groups, no significant changes were detected at 1 month (*p* = 0.571) as well as at 6 months of diet intervention (*p* = 0.828). However, at the end of the 6 months, mice that were fed a standard diet and those fed an LSO diet had their scores reduced by 12.5% each, significantly below the chance level (one-sample *t*-test *p* < 0.048), suggesting a working memory deficit. Mice that were fed an FO diet also had their alternation score reduced in a similar way (12.5%) but stayed at chance level (50%), while those that were fed Schizo or DHASCO diets maintained their scores. All dietary groups had choice latencies below 40 s in the sample trial (Figure 1K), with no significant differences among groups (*p* = 0.422). Test trial latencies (Figure 1L) were also similar across groups (*p* = 0.503). These results suggest no motor or anxiety differences between the studied groups and so do indicate that the observed exploratory behaviour was not due to mobility impairments or a lack of motivation. These findings suggest that spatial working memory is protected in mice fed FO, Schizo, or DHASCO diet, while a cognitive deficit is evident in standard and LSO groups. Overall, incorporating FO, Schizo or DHASCO oils into the dietary routine shows promising protective effects on the working memory of 5×FAD mice following six months of nutritional intervention.

### 3.3. Plasma Biochemistry Profile

Plasma metabolites in 5×FAD mice were largely influenced by the experimental diets, as seen in Table 2. Although insulin concentration was below the detection limit of the equipment (<0.4 mU/L), glucose levels were significantly increased in mice that were fed DHASCO oil compared to the other diets (*p* < 0.001). The FO diet also increased urea levels relative to the Schizo diet (*p* = 0.035). Creatinine levels were higher in the control mice relative to DHASCO oil-fed mice (*p* = 0.044). Cholesterol levels were increased in reference mice relative to all the other dietary groups, except for DHASCO oil-fed mice (*p* < 0.001). The variation in HDL cholesterol (*p* < 0.001) and total lipids (*p* < 0.001) were identical to total cholesterol. LDL cholesterol reached the highest levels in the FO diet, when compared to Schizo oil, DHASCO oil, and reference mice (*p* < 0.001). VLDL cholesterol and triacylglycerols were found reduced in the FO diet when compared to the LSO diet (*p* = 0.013). For the hepatic markers, the control diet increased ALT relative to LSO and DHASCO diets (*p* = 0.003). AST levels were increased in mice fed FO relative to the other diets (*p* < 0.001), but GGT was found unchanged (*p* > 0.05). Total protein levels were kept similar amongst dietary groups (*p* > 0.05). The acute phase protein, C-reactive protein, reached the highest values in mice fed LSO compared to the other diets (*p* < 0.001). Concerning the inflammatory status, both IGF-1 (<7.00 µg/L) and IL-6 (<1.5 ng/L) were below the minimum detection limit, suggesting no inflammation in 5×FAD mice from this nutritional assay.

#### 3.3.1. Pearson Correlation Coefficients Amongst Plasma Metabolites

We next performed a correlational analysis using Pearson correlation coefficients to find positive or negative, strong or weak, associations between plasma metabolites. Glucose was negatively correlated with creatinine (r = −0.338, *p* = 0.035) and with ALT (r = −0.378, *p* = 0.016). Urea was moderately correlated with AST (r = 0.379, *p* = 0.016). Creatinine was negatively correlated with triacylglycerols (r = −0.395, *p* = 0.013) and with VLDL cholesterol (r = −0.395, *p* = 0.012). Total cholesterol was negatively correlated with LDL cholesterol (r = −0.333, *p* = 0.036) and AST (r = −0.313, *p* = 0.049) but strongly positively with HDL cholesterol (r = 0.921, *p* < 0.001) and total lipids (r = 0.971, *p* < 0.001). LDL cholesterol was inversely correlated with HDL cholesterol (r = −0.492, *p* = 0.001) and total lipids (r = −0.357, *p* = 0.024). HDL cholesterol was further correlated positively with ALT (r = 0.410, *p* = 0.009) and total lipids (r = 0.872, *p* < 0.001) but negatively with AST (r = −0.343, *p* = 0.030). Triacylglycerols were negatively correlated with AST (r = −0.506, *p* = 0.001) and total protein (r = −0.350, *p* = 0.027) but highly positively with VLDL cholesterol (r = 1, *p* < 0.001) and slightly positively with total lipids (r = 0.312, *p* = 0.049). AST presented negative correlations with VLDL cholesterol (r = −0.506, *p* = 0.001), PCR (r = −0.461, *p* = 0.003), and total lipids (r = −0.420, *p* = 0.007). GGT was uncorrelated with any plasma metabolite parameter (*p* > 0.05). Total proteins were also negatively correlated with VLDL cholesterol (r = −0.349, *p* = 0.027). VLDL cholesterol was also marginally correlated with total lipids (r = 0.312, *p* = 0.049).

#### 3.3.2. Principal Component Analysis Using Plasma Metabolites

**Figure 2:**
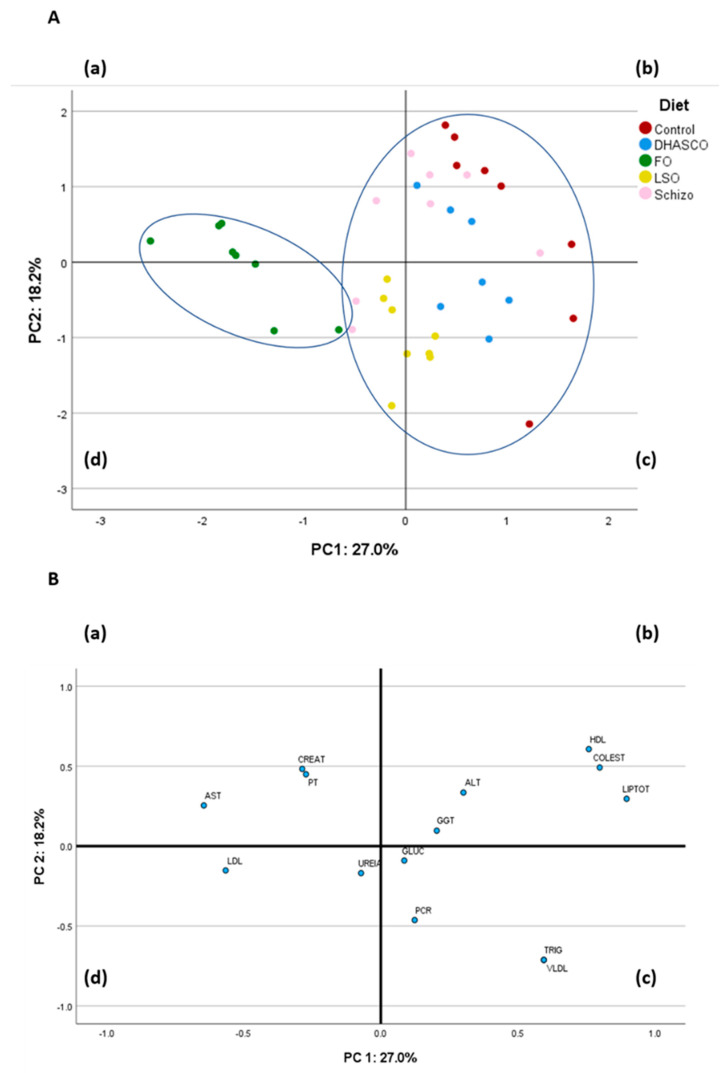
Loading plot of the first and second principal components (PCs) of the component score vectors (**A**) and pooled data (**B**) for plasma metabolites in 5×FAD mice that were fed the following: control diet; LSO, linseed oil diet; FO, cod liver oil diet; Schizo, *Schizochytrium* microalga oil diet; and DHASCO, commercial DHASCO oil diet. Blue circles indicate the discrimination of dietary groups.

To conclude the analysis of plasma biochemical profile, we next performed a principal component analysis (PCA) having 14 variables in the correlation matrix (Figure 2). The PCA using plasma metabolites was performed to describe the variability of the pooled data into two dimensions (Figure 2B). The score plot of the first two PC explained 45.2% of the total variability, with 27.0% for PC1 and 18.2% for PC2. The PC1 was characterized by variables with positive loadings, such as total lipids (0.897), cholesterol (0.799), HDL cholesterol (0.759), triacylglycerols (0.595), and VLDL cholesterol (0.595), and by variables with negative loadings, such as AST (−0.646) and LDL cholesterol (−0.566). Concerning the PC2, it was characterized by variables with positive loadings, such as HDL cholesterol (0.607), cholesterol (0.492), creatinine (0.483), and total proteins (0.449), and by variables with negative loadings, such as triacylglycerols (−0.712), VLDL cholesterol (−0.712), and C-reactive protein (−0.463). The score plot depicted in Figure 2A shows the location of the five experimental groups in the multivariate space of the first two PC. The discrimination of dietary treatments was attainable with the fish oil diet clearly separated from the other diets (Figure 2A). The control and DHASCO oil groups were located in quadrants (b) and (c), whereas the LSO group was located in quadrants (c) and (d) (Figure 2A). The Schizo diet was dispersed across quadrants (a), (b), and (d) (Figure 2A).

### 3.4. Neuronal Count and Immunohistochemistry Staining in Mouse Brains

**Figure 3:**
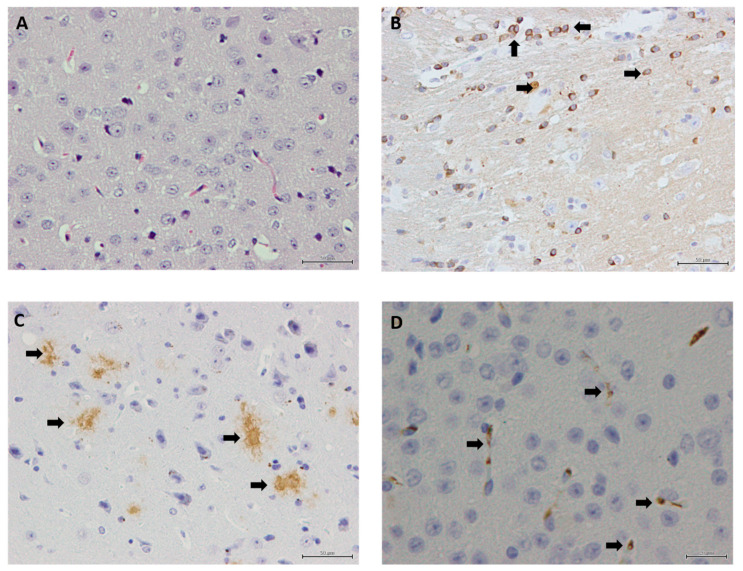
Illustrative images of H&E ((**A**), magnification ×200, scale bar = 50 μm) staining and immunohistochemistry markers for TAU ((**B**), magnification ×200, scale bar = 50 μm), β-amyloid plaques ((**C**), magnification ×200, scale bar = 50 μm), and IBA1 ((**D**), magnification ×400, scale bar = 20 μm) marked with arrows in the cerebral cortex of 5×FAD mice.

**Figure 4:**
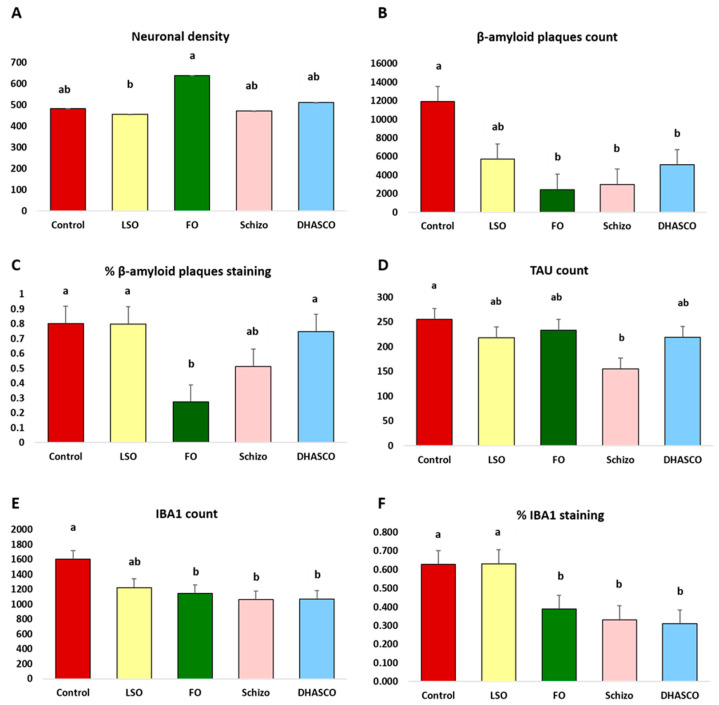
Neuronal density by H&E staining (**A**) and immunohistochemistry markers for β-amyloid plaque count (**B**) and staining area (**C**), TAU count (**D**), and IBA1, count (**E**) and staining area (**F**) in the cerebral cortex of 5×FAD mice fed the following diets: control diet; LSO, linseed oil diet; FO, cod liver oil diet; Schizo, *Schizochytrium* microalga oil diet; and DHASCO, commercial DHASCO oil diet. In total, 20 photos/fields were used per section at ×400 magnification (real area of each photo at ×400 magnification = 23,170 μm^2^). IBA1: Ionized calcium-binding adapter molecule 1. Data are mean ± SEM (standard error of the mean). Different letters indicate a significant difference (*p* < 0.05).

Figure 3 shows illustrative images in the cerebral cortex of 5×FAD mice stained with hematoxylin and eosin (H&E) (Figure 3A) and with immunohistochemistry markers for TAU (Figure 3B), β-amyloid plaques (Figure 3C), and IBA1 (Figure 3D). Figure 4 depicts the associated neuronal density (Figure 4A) and β-amyloid plaque (Figure 4B,C) count and area, respectively, TAU (Figure 4D), and IBA1 (Figure 4E,F) count and area, respectively, in the cerebral cortex of 5×FAD mice.

H&E staining was applied to evaluate neuronal density, with identical neurons count across diets, except for the fish oil diet with an increased number (*p* = 0.031) relative to linseed oil (Figure 4A). From less neurons to more neurons, LSO ^b^ < Schizo ^ab^ < Control ^ab^ < DHASCO ^ab^ < FO ^a^. Immunohistochemistry using antibodies directed against β-amyloid plaques showed that DHA-enriched diets promoted reduced plaque count (*p* = 0.002) relative to reference mice (Figure 4B), while smaller β-amyloid plaques were positively observed only in the fish oil diet (*p* = 0.007) (Figure 4C). Immunostaining with TAU antibody showed a reduced number of TAU-positive cells in mice that were fed a Schizo diet relative to the reference mice (*p* = 0.028) with a pattern of variation from minus to plus, like this: Schizo ^b^ < LSO ^ab^ < DHASCO ^ab^ < FO ^ab^ < Control ^a^ (Figure 4D). The histological detection of IBA1, a sensitive marker of CNS microglial, whose expression is increased with microglia activation, showed significant variations for the number of IBA1-positive cells (*p* = 0.012) (Figure 4E) and area (*p* = 0.002) (Figure 4F) amongst dietary groups, both parameters reduced in DHA-enriched diets.

#### Principal Component Analysis Using Histological and Immunohistochemistry in the Brain

**Figure 5:**
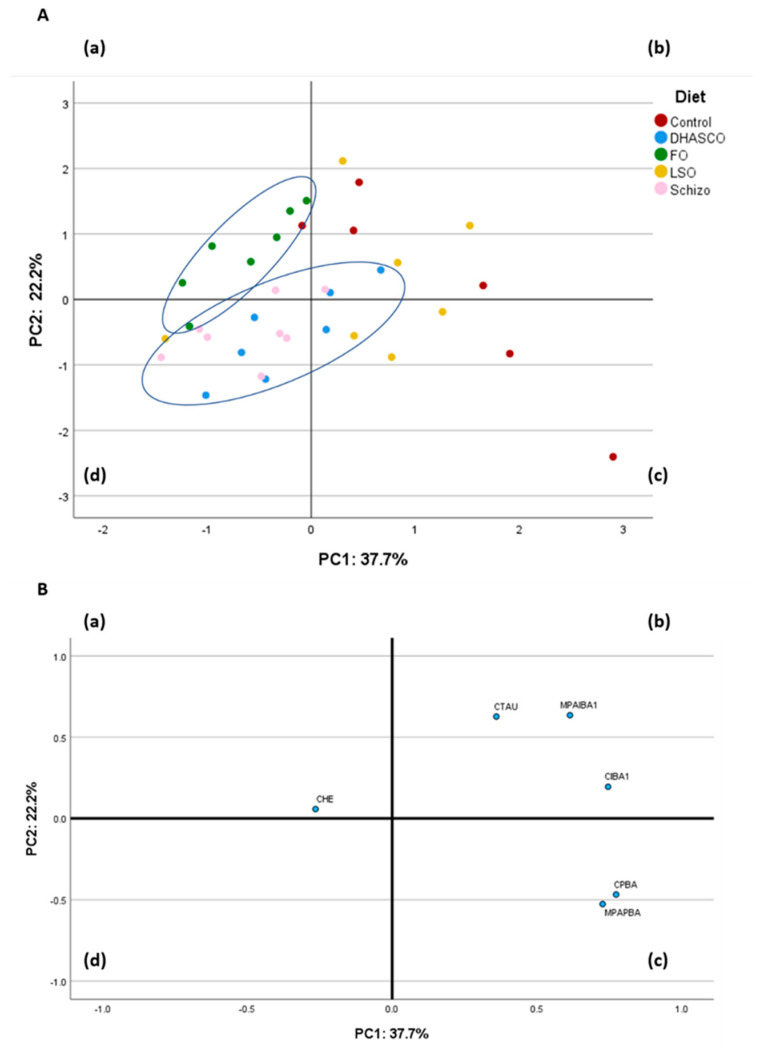
Loading plot of the first and second principal components (PC) of the component score vectors (**A**) and pooled data (**B**) for histological and immunohistochemistry markers in the brain of 5×FAD mice fed the following diets: control diet; LSO, linseed oil diet; FO, cod liver oil diet; Schizo, *Schizochytrium* microalga oil diet; and DHASCO, commercial DHASCO oil diet. Blue circles indicate the discrimination of dietary groups.

We next performed a PCA having six immunohistochemistry-associated variables in the correlation matrix (Figure 5). The PCA using histological and immunohistochemistry markers was performed to describe variability of the pooled data into two dimensions (Figure 5B). The score plot of the first two PC explained 59.9% of the total variability, with 37.7% for PC1 and 22.2% for PC2. The PC1 was characterized by variables with positive loadings, such as β-amyloid plaque count (0.774), β-amyloid plaque staining area (0.727), IBA1 count (0.746), and IBA1 staining area (0.614). Concerning the PC2, it was characterized by variables with positive loadings, such as the IBA1 staining area (0.635) and TAU count (0.626), and by variables with negative loadings, such as the β-amyloid plaque staining area (−0.526) and β-amyloid plaque count (−0.469). The score plot depicted in Figure 5A shows the location of the five experimental groups in the multivariate space of the first two PC. The discrimination of dietary treatments was attainable with the FO diet concentrated mostly in quadrant (a) and clearly separated from the other diets (Figure 5A). In addition, the Schizo and DHASCO oil groups were visibly concentrated in one cluster covering all quadrants (Figure 5A). The control group was located in quadrants (a), (b), and (c), whereas the LSO group was more dispersed and located in quadrants (b), (c), and (d) (Figure 5A).

### 3.5. Fatty Acid Profile in Mice Liver

Table 3 presents the detailed fatty acid composition in the liver of mice fed the experimental diets.

The hepatic fatty acid profile was largely influenced by DHA-enriched diets. Except for mice that were fed the Schizo diet, the sum of saturated fatty acids (SFAs) was increased in mice that were fed DHASCO oil relative to the other dietary groups (*p* < 0.001). This increase was mostly due to changes in palmitic acid (16:0) (*p* < 0.001) contents. Conversely, the sum of monounsaturated fatty acids (MUFAs) was higher in all dietary groups (*p* < 0.001) relative to DHASCO, at the expense of 16:1n-9 (*p* = 0.003), 16:1n-7 (*p* < 0.001), 18:1n-9 (*p* = 0.001), and 18:1n-7 (*p* < 0.001) fatty acids. Total PUFAs were increased in mice that were fed DHASCO oil relative to control and fish oil (*p* = 0.001) diets, mainly due to changes observed for n-3 PUFAs (*p* < 0.001), in particular for DHA (*p* < 0.001), which stands for almost the totality of n-3 PUFAs identified in mice liver. EPA levels were identical in mice fed n-3 fatty acid-enriched diets when compared to the reference group (*p* < 0.001). The sum of n-6 PUFAs was kept unchanged across diets (*p* = 0.066), mostly at the expense of linoleic acid (18:2n-6) (*p* = 0.874), which did not vary. It should also be noted that arachidonic acid (20:4n-6) was enhanced in mice that were fed the control diet when compared to the others (*p* < 0.001).

Following the changes described for n-3 and n-6 PUFA, the n-3/n-6 ratio reached the highest levels in mice that were fed DHASCO oil relative to the other diets and the lowest levels in reference mice (*p* < 0.001).

#### Principal Component Analysis Using Fatty Acid Sums and n-3/n-6 Ratio in the Liver

**Figure 6:**
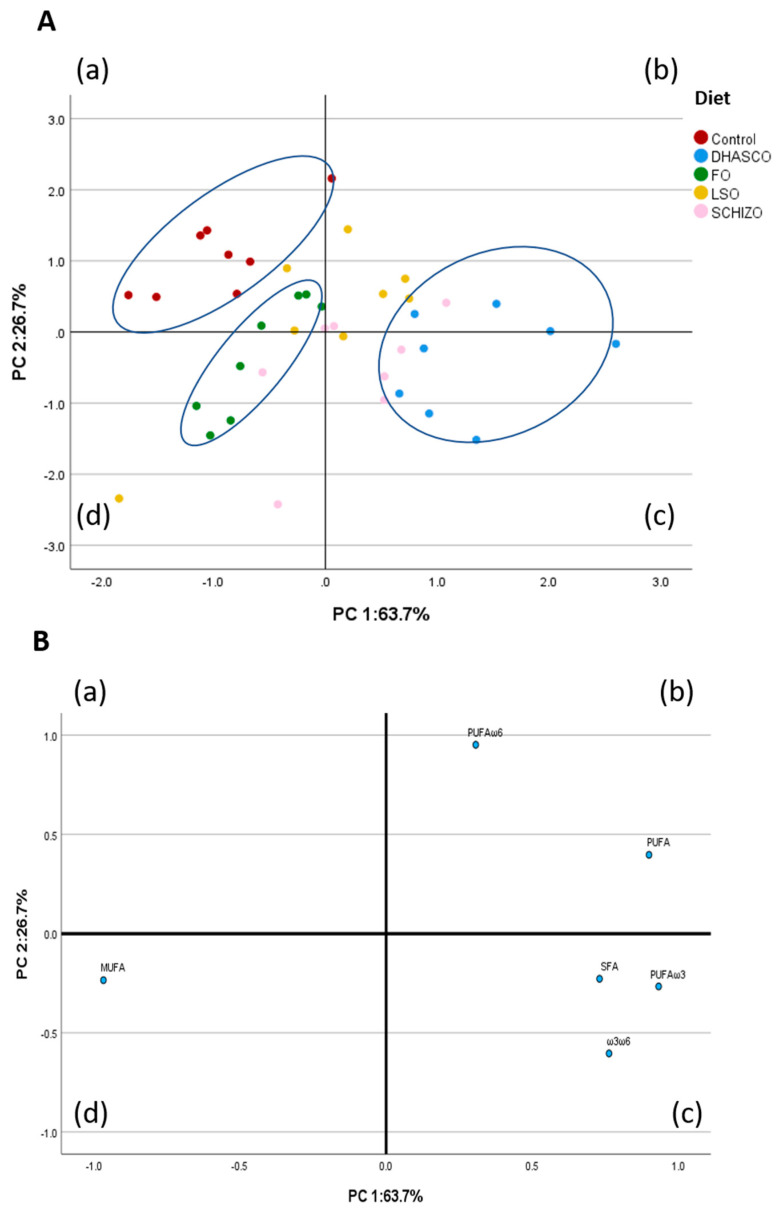
Loading plot of the first and second principal components (PCs) of the component score vectors (**A**) and pooled data (**B**) for the fatty acid sums and n-3/n-6 ratio in the liver of 5×FAD mice fed the following diets: control diet; LSO, linseed oil diet; FO, cod liver oil diet; Schizo, Schizochytrium microalga oil diet; and DHASCO, commercial DHASCO oil diet. Blue circles indicate the discrimination of dietary groups.

A PCA was then performed using hepatic fatty acid sums and n-3/n-6 ratio in the correlation matrix (Figure 6) up to a total of six variables. The PCA using fatty acid sums and n-3/n-6 ratio in the liver was performed to describe variability of the pooled data into two dimensions (Figure 6B). The score plot of the first two PC explained 90.4% of the total variability, with 63.7% for PC1 and 26.7% for PC2. The PC1 was characterized by variables with positive loadings, such as n-3 PUFA (0.933), total PUFA (0.900), n-3/n-6 PUFA ratio (0.763), and SFA (0.730), and by variables with negative loadings, such as MUFA (−0.967). Concerning the PC2, it was characterized by variables with positive loadings, such as n-6 PUFA (0.951), and by variables with negative loadings, such as the n-3/n-6 PUFA ratio (−0.604). The score plot depicted in Figure 6A shows the location of the five experimental groups in the multivariate space of the first two PCs. The discrimination of dietary treatments was attainable, in particular for the control, FO, and DHASCO oil diets. The control diet concentrated mostly in quadrant (a) and was clearly separated from the other diets (Figure 6A). The FO diet was concentrated in quadrants (a) and (d), whereas the DHASCO oil group was concentrated in quadrants (b) and (c) (Figure 6A). The Schizo diet was dispersed across quadrants (b), (c), and (d) (Figure 6A), while the LSO group was scattered across all four quadrants (Figure 6A).

### 3.6. Fatty Acid Profile in Mouse Brains

The entire hemi-brain was used for the determination of the fatty acid composition in the brain of mice that were fed the experimental diets. Table 4 presents the detailed fatty acid composition in the brain of mice that were fed the experimental diets. Major variations were found across fatty acids in the brain under the influence of DHA-enriched diets. The sum of saturated fatty acids (SFAs) did not vary across diets (*p* = 0.637), mostly due to the non-variations in predominant fatty acids, such as palmitic acid (16:0) (*p* = 0.732) and stearic acid (18:0) (*p* = 0.477). In a similar trend, the sum of monounsaturated fatty acids (MUFAs) was not influenced by diets (*p* = 0.182), reflecting the non-variations observed for 16:1n-7 (*p* = 0.271) and 20:1n-9 (*p* = 0.639) fatty acids but not for 16:1n-9 (*p* = 0.001), 18:1n-9 (*p* = 0.029), and 18:1n-7 (*p* < 0.001) fatty acids. As far as it concerns 16:1n-9, it reached higher levels in the LSO and FO groups in comparison to Shizo and DHASCO diets (*p* = 0.001). 18:1n-9 was increased in the Schizo diet compared to the reference mice (*p* = 0.029), while 18:1n-7 presented the inverse trend (*p* < 0.001). The sum of PUFAs was reduced in mice fed fish oil relative to reference mice (*p* = 0.014). The changes observed for n-3 PUFA sum (*p* < 0.001) are related to statistically higher contents for Schizo and DHASCO oils, intermediate contents for fish oil, and lower contents for control and LSO diets, reflecting the variations observed for DHA (*p* < 0.001), which represented almost the totality of n-3 PUFAs identified in mouse brains. 16:3n-3 and 16:4n-3 presented similar brain contents across diets (*p* > 0.05 for both). 18:3n-3 was undetected in mouse brains. The values for 20:5n-3 were increased in Schizo and DHASCO oil diets, reduced in LSO and FO diets, and not detectable in the control group (*p* < 0.001). 22:5n-3 was found increased in n-3 PUFA-enriched diets relative to the reference mice (*p* < 0.001). On the contrary, total n-6 PUFA was reduced in mice that were fed LSO and DHA-enriched oils compared to the reference (*p* < 0.001). This significant reduction reflects largely the changes observed for 20:4n-6 (*p* < 0.001) and 22:4n-6 (*p* < 0.001) fatty acids but not those observed for 18:2n-6 (*p* = 0.008) and 22:5n-6 (*p* < 0.001) fatty acids. Linoleic acid (18:2n-6) reached the highest contents in linseed oil and DHA-enriched diets in comparison to the reference group (*p* = 0.008), whereas 22:5n-6 fatty acid was absent in LSO and fish oil dietary groups but higher in DHASCO, intermediate in Schizo, and lower in the reference group (*p* < 0.001).

Following the variations observed for the sum of n-3 and n-6 PUFA, the n-3/n-6 ratio was increased with a similar content in mice that were fed Schizo and DHASCO oils, compared to FO, LSO, and reference diets (*p* < 0.001).

#### Principal Component Analysis Using Fatty Acid Sums and n-3/n-6 Ratio in the Brain

**Figure 7:**
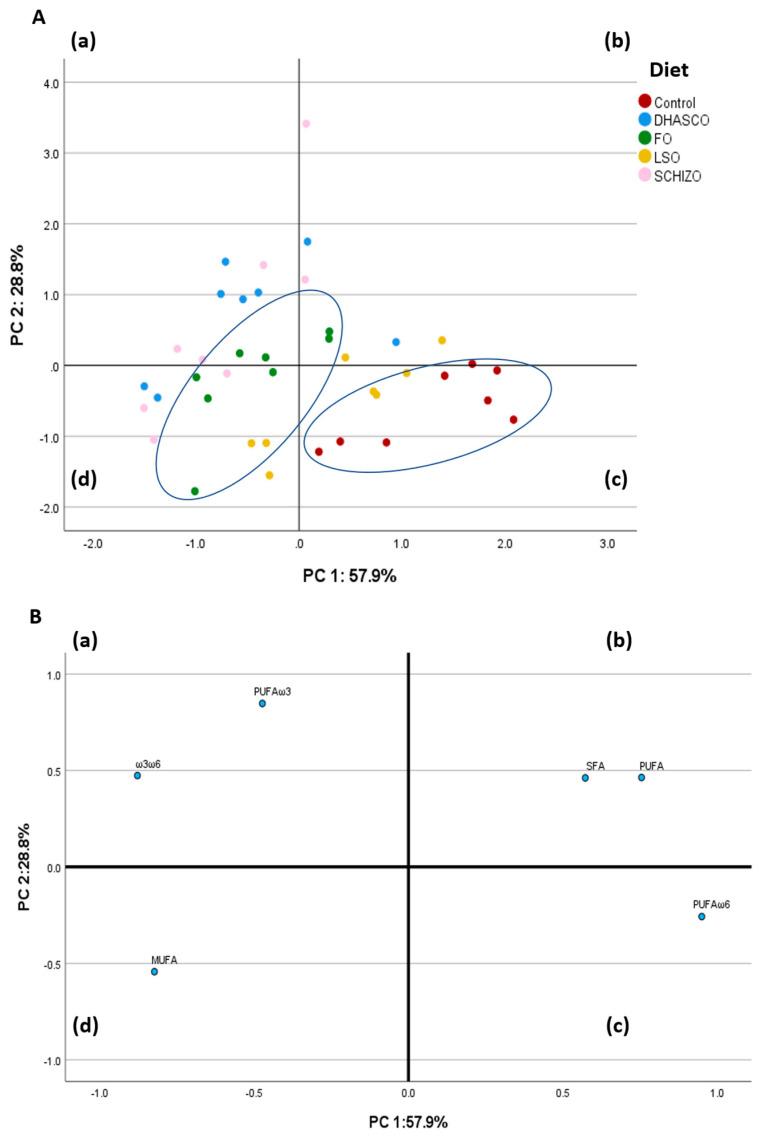
Loading plot of the first and second principal components (PC) of the component score vectors (**A**) and pooled data (**B**) for the fatty acid sums and n-3/n-6 ratio in the brain of 5×FAD mice fed: control diet; LSO, linseed oil diet; FO, cod liver oil diet; Schizo, *Schizochytrium* microalga oil diet; and DHASCO, commercial DHASCO oil diet. Blue circles indicate the discrimination of dietary groups.

We next performed a PCA using brain fatty acid sums and the n-3/n-6 ratio in the correlation matrix (Figure 7) up to a total of six variables. The PCA using fatty acid sums and the n-3/n-6 ratio in the brain was performed to describe variability of the pooled data into two dimensions (Figure 7B). The score plot of the first two PC explained 86.7% of the total variability, with 57.9% for PC1 and 28.8% for PC2. The PC1 was characterized by variables with positive loadings, such as n-6 PUFA (0.950), total PUFA (0.755), and SFA (0.573), and by variables with negative loadings, such as the n-3/n-6 ratio (−0.878) and MUFA (−0.823). Concerning the PC2, it was characterized by variables with positive loadings, such as n-3 PUFA (0.848), and by variables with negative loadings, such as MUFA (−0.542). The score plot depicted in Figure 7A shows the location of the five experimental groups in the multivariate space of the first two PC. The discrimination of dietary treatments was attainable, in particular for the control and FO diets. The control diet concentrated mostly in quadrants (b) and (c), while the FO diet was located in quadrants (a), (b), and (d) (Figure 7A). Like FO, the DHASCO oil and the Schizo diet groups were also scattered across quadrants (a), (b), and (d), while the LSO group was scattered across quadrants (b), (c), and (d) (Figure 7A).

## 4. Discussion

Diet is one of the most important exposures that may affect health throughout a lifespan. Besides studying novel pharmacological targets for drug development and discoveries that may solve AD epidemic health conditions, scientists also strive to find promising components of a dietary strategy for preventing AD through the pivotal role of their key nutrients in neuroprotection.

In this study, after 6 months of nutritional intervention, diet had no impact on final body weight, body weight gain, or average daily feed intake of 5×FAD mice. These results suggest that DHA-enriched diets did not influence energy intake or body weight variations, suggesting no effect on the overall caloric balance or mice growth.

In terms of average daily feed intake of DHA and as expected, mice that were fed the control diet and linseed oil had no intake of DHA per day. Mice that were fed fish oil consumed daily around 110 ± 7 mg of DHA. Mice that were fed *Schizochytrium* microalga oil and DHASCO oil ingested approximately 376 ± 23 mg and 595 ±39 mg of DHA per day, respectively. Therefore, the daily ingestion of DHA by 5×FAD mice from different dietary treatments reflects entirely the nutritional chemical composition of experimental diets.

Blood metabolites often fingerprint dietary patterns [48,49]. Herein, mice that were fed DHASCO oil exhibited increased glucose levels relative to the other diets, even though their insulin concentrations remained below the detection limit, suggesting no departure from glucose homeostasis. In line with this, no changes in the final body weight of mice were observed. Markers for renal function presented small changes for urea and creatinine across diets, all of them within reference figures for 5×FAD mice and, therefore, devoid of any clinical physiological relevance.

Under these experimental settings, blood lipemia was largely influenced by diets. Except for LDL cholesterol and triacylglycerols, reference mice had increased levels of total cholesterol, HDL cholesterol, and total lipids, demonstrating that our diet formulations with n-3 PUFA reduced most of the blood lipid parameters. Concerning the hepatic markers, the increased levels of ALT in reference mice and AST in mice that were fed fish oil, alongside the maintenance of GGT, suggest that the hepatic homeostasis was maintained in 5×FAD mice despite these minor metabolic changes. A striking reduction in C-reactive protein, an acute phase protein of liver origin [50], in DHA-enriched diets, compared to the LSO group, suggests the existence of potential anti-inflammatory effects when lipid diets are compared. Total protein was unaffected by dietary regimens. Additionally, IGF-1 and IL-6 levels in the plasma were below the detection limits, pointing towards the non-existence of systemic inflammation across all diets. These findings support the evidence that dietary patterns, such as the one provided by DHA-enriched diets, are fingerprinted by blood metabolites, re-enforcing the discriminant analysis of plasma metabolites that did set apart the fish oil group from the others.

The liver plays a key role in lipid metabolism [51,52]. Depending on the species, it is, more or less the hub of fatty acid synthesis and lipid circulation through lipoprotein synthesis [52]. In this study, hepatic fatty acid composition was found to be dependent on DHA-enriched diets. The most striking finding was that the total PUFA found increased in mice that were fed DHASCO oil relative to fish oil or control diets, mainly due to changes observed for n-3 PUFA, in particular for DHA, which accounts for almost all of the total n-3 PUFA identified in mice liver. Hepatic EPA levels were identical in mice fed n-3 fatty acid-enriched diets. Another positive finding was that the n-6 PUFA sum was unchanged across diets, mostly at the expense of linoleic acid (18:2n-6), which did not vary. The pro-inflammatory fatty acid arachidonic acid (AA, 20:4n-6) gives rise to eicosanoids and other bioactive lipid mediators with established roles in inflammation, with AA metabolism a recognized target for commonly used anti-inflammatory therapies [53,54]. In here, AA levels were found reduced across all n-3 LCPUFA-supplemented diets. As a consequence, the n-3/n-6 ratio reached the highest levels in the liver of mice fed DHASCO oil and the lowest levels in reference mice.

Among n-3 LCPUFA, DHA is vital for brain development and functionality, protecting against age-related cognitive decline [3]. DHA plays a key role in neurogenesis, neuroplasticity, and neuron health [7], which are essential for synaptic transmission, learning, and memory [5]. Moreover, a lack of dietary n-3 fatty acids can prevent the renewal of membranes and thus accelerate cerebral ageing [55]. In this study, the brain fatty acid profile of 5×FAD mice, as seen already in the liver, reflects their dietary intake according to the treatment group, which totally agrees with the revised literature [56,57,58]. Indeed, based on the fatty acid composition, the statistical discriminant tool clearly discriminated the reference, fish oil, and DHASCO oil-fed mice in the liver, as well as the reference and the fish oil-fed mice in the brain. Nonetheless, the brain’s fatty acid composition is more conservative and, therefore, less responsive to changes in dietary fatty acid intake compared to the liver [57,59,60]. Dietary supplementation with *Schizochytrium* sp. microalga and DHASCO oils increased DHA levels in the brain relative to the reference group and linseed oil group but was not different from the fish oil group, while EPA was residual in numeric terms, likely due to its conversion into DHA [61]. DHA makes up over 90% of the n-3 PUFAs in the brain and 10–20% of its total lipids [62]. The n-3/n-6 fatty acid ratio was particularly favourable in the brain of mice that were fed *Schizochytrium* sp. microalga oil and DHASCO oil, even surpassing fish oil, just like in the liver, highlighting the beneficial effects of these DHA-enriched diets on brain lipid metabolism, which is often altered in AD. These results suggest that the sustained cognitive function observed in *Schizochytrium* sp. microalga and DHASCO oils groups may be attributed to the anti-inflammatory and neuroprotective effects of high DHA doses [3,4,28,29]. In fact, among the PUFA analyzed, DHA exhibited the largest number of double bonds and was found to be the most potent anti-inflammatory compound [63]. Conversely, our research team reported a few years ago that the combination of DHA with EPA from fish oil counteracts the undesirable health effects of saturated fat-based diets and benefit, in the long run, neurological function in Wistar rats, a healthy animal model, overcoming microalgae oils [30,32], and other terrestrial plant oils [31,32] enriched with these n-3 LCPUFA.

Both humans and murine models display synaptic degeneration, but the severity is generally milder in animal models. This feature correlates with cognitive impairment and is better replicated in specific models, such as the one selected by us in this study, the 5×FAD mice [64,65]. Cognitive impairment and decline are key features in human AD, impacting memory and daily functions. Murine models also display memory deficits but usually in simplified forms, assessed with behavioural tasks, like the Morris water maze test or the T-Maze test [66,67], the latter selected for our study.

Our data suggest that DHA from sustainable natural sources, such as the *Shizochytrium* sp. microalga (including the DHASCO oil diet), might convey twice the protection in the AD context, by alleviating cognitive impairment, exerting its beneficial action. Algae-derived dietary supplementation mitigated the decline in working memory and recognition memory observed in 5×FAD mice after six months of dietary supplementation. Other studies have also demonstrated that 5×FAD mice exhibit memory deficits and learning impairment as early as 1–2 months old, as assessed by the Morris water maze test [68,69]. Other authors report cognitive impairment from 4 months old, as assessed by the Morris water maze, Y-maze, and olfactory H-maze tests [38,70,71].

DHA plays a pivotal role in neurogenesis, synaptic transmission, and anti-inflammatory action. Furthermore, DHA enhances cellular membrane fluidity and permeability by modulating the rigidity of the cell membrane [72,73], interferes with different signalling pathways [74,75,76], and reduces pro-inflammatory marker secretion [63,77,78,79]; all of these mechanisms work together and positively impact neuroprotection.

The 5×FAD mice are currently one of the most used experimental animal models for AD research and therefore one of the best characterized, co-expressing five FAD mutations, in which three mutations are located in the APP gene, and the other two mutations are located in the PS1 gene [10,22]. These mice display strong amyloid pathology with enhanced levels of β-amyloid and intraneuronal deposition from 1.5 months old and initial formation of extracellular deposits from 2 months old, primarily in the subiculum, deep layers of the cortex and the frontal cortex with an age-dependent progression, affecting in due course much of the cortex, subiculum, and hippocampus areas [38,70,80,81,82]. Neuroinflammation, evaluated by reactive astrocytes and microglia, is observed at 2 months old in parallel with extracellular β-amyloid deposition, with age-dependent progression as well as identical distribution and localization [38,70,81,82]. The synaptic transmission impairment at 4-month-old mice concurs with the beginning of neuronal decline and aggravates neuronal loss around 9 months old [70,80,82,83].

The histological analysis of brain lesions in the cerebral cortex of 5×FAD mice through classical H&E staining and immunohistochemistry technique showed significant differences across diets concerning Aβ deposition, TAU protein, or IBA1. The neuronal density was found to be higher in mice that were fed fish oil relative to those fed linseed oil. Since no other changes were observed in the remaining DHA-enriched diets from distinct origins, this variation might be attributed to the synergetic effects of DHA combined with EPA in cod liver oil that are not found in DHASCO or *Schizochytrium* sp. microalga oil diets [30].

Gliosis, featured by reactive astrocytes and microglia clustered around dense-core amyloid plaques, is a hallmark of AD [84,85,86]. IBA1 is a well-established marker of macrophages/microglia [87,88], whose expression is increased with its activation. IBA1 polyclonal antibody detects IBA1 protein at the cell membrane by immunohistochemistry analysis. The observed reduced IBA1 staining (both area and number) across the cerebral cortex of mice that were fed DHA-enriched diets is consistent with reduced microgliosis, which could result in reduced neuroinflammation, as observed by other authors [89] (but that was not independently measured in the present study). These results suggest that dietary treatments significantly altered the progression of specific AD-associated brain lesions. Also, the decreased brain content in the pro-inflammatory AA (20:4n-6) precursor of eicosanoids with established roles in inflammation is suggestive of a lower neuroinflammation setting in those animals fed DHA-rich diets [53,54]. Although neuroinflammation is seen in both humans and mice, the immune response differs. Murine models show glial activation, but this is less extensive than in humans, where chronic inflammation drives further damage [90,91]. Taken together, our findings reveal that the chemical composition and concentration of dietary FA are key factors in the intricate regulation of mediated inflammation [63]. This outcome may seem consistent with the observed benefits of Schizo and DHASCO oil diets on 5×FAD mouse cognitive function. Aβ itself may be considered the primary chemotactic signal for activated microglia, whereas astrocytes might primarily respond to plaque-associated neuritic damage [85]. Immunohistochemistry analysis of β-amyloid in a formalin-fixed paraffin-embedded transgenic mouse brain that expresses FAD mutant APP and presenilins (PSEN1 and PSEN2) shows strong cytoplasmic staining in amyloid plaques, which was diet-dependent. Our tested DHA-enriched diets reduced plaque count, and fish oil also reduced the plaque area in line with the observed IBA1 reduction, suggestive of decreased neuroinflammation. Like our results, DHA significantly reduced the deposition of Aβ in the brain [92,93] and inhibited the production of nerve fibres, thereby increasing cognitive abilities in AD [93]. Murine models, typically genetically engineered to express APP mutations, develop amyloid plaques. However, plaque composition and regional distribution often differ from those in human AD, where amyloid-beta (Aβ42) plaques accumulate in the hippocampus and cortex, significantly impacting neurodegeneration [94,95].

TAU is a neuronal microtubule-associated protein found predominantly on axons whose function is to promote tubulin polymerization and stabilize microtubules [96,97]. Besides AD, hyperphosphorylated TAU is found in neurofibrillary lesions in a range of other central nervous system disorders, such as Pick’s disease or frontotemporal dementia [98,99]. Herein, the measurement of total TAU levels (rather than hyperphosphorylated TAU, which was not measured in this study) acts as an indicator of TAU accumulation. The 5×FAD mice do not normally develop TAU pathology, including neurofibrillary tangles and neuritic plaques [82,100,101]. Moreover, 5×FAD mice have the potential to develop TAU pathology following injection with human TAU, or when TAU is overexpressed, and there is some evidence that TAU aggregates develop in 5×FAD brains [102], thus corroborating our findings. In this study, the immunohistochemistry analysis of TAU showing staining in the cytoplasm and membrane of paraffin-embedded mouse brain tissue revealed a reduced number of TAU-positive cells in mice that were fed a Schizo diet relative to the reference mice. The neuroprotective effects of DHA from sustainable origins primarily support synaptic function and neuroplasticity by directly altering or reducing amyloid plaques that were already established as well as TAU accumulation in the brain. Overall, the improved discrimination in the multivariate space observed for dietary groups incorporating fish oil suggests a beneficial protection on brain lesions in 5×FAD mice and may reflect enhanced neuronal resilience occurring at the traditional hallmarks of AD pathology, while diets supplemented with high DHA doses, like *Schizochytrium* sp. microalga and DHASCO oils, attenuated significantly AD manifestations in terms of the amyloid plaque number or TAU accumulation and associated microgliosis, offering functional benefits that may contribute to the preservation of cognitive function. As such, DHA promoted Aβ clearance and reduced Aβ aggregation as well as TAU-stained positive cells, both of which are recognized features of AD pathophysiology. Even if evident variations in these features were observed herein, a clear conservation of cognitive behaviour was depicted. The improved cognitive function observed in our study might be associated with the modulation of the BDNF (brain-derived neurotrophic factor), a neurotrophin vital for neurogenesis, neuroplasticity, and neuroprotection, known to exert a key role in cognitive function [30,31,103,104], which unfortunately was not measured by us. Several studies have demonstrated that BDNF levels increased in the brain by fish- or krill oil-enriched diets [103,104]. The exact mechanism underlying this cause–effect relationship remains uncertain although some reports suggest a direct effect of DHA on the transcriptional profile of the BDNF [104]. Another possible mechanistic explanation for the improvement of brain function is related to the fact that DHA induces the activity of antioxidant enzymes [103,105,106], positively impacting redox status maintenance. If BDNF levels are negatively correlated with oxidative stress [103], the reduction in oxidative stress will promote an increase in BDNF levels.

All together, these findings support the evidence that optimal nutrition, such as the one provided by DHA-enriched diets, is key for brain function and for the prevention of neurodegenerative diseases [107,108,109].

## 5. Conclusions

The simple modification of lifestyle and health behaviours, such as diet, is a particularly effective public health target for neurodegenerative disease prevention, including AD. Such a novel insight offers potential options for improved treatment. In fact, incorporating *Schizochytrium* sp. microalga and DHASCO commercial oils into dietary routines, at an approximately human equivalent dose of 0.88 g/kg and 1.44 g/kg of DHA, respectively, shows promising protective effects on the working- and the recognition–memory function of 5×FAD mice following six months of nutritional intervention which is concurred with DHA brain enrichment. The fish oil, at human equivalent dose of 0.24 g/kg of DHA, appears to have protective effects on the working memory function of 5×FAD mice and has showed remarkably powerful beneficial variations in brain lesions of 5×FAD mice, highlighting synaptic plasticity, in addition to features also seen in high-DHA-dose diets, such as the clearance of β-amyloid and reduced neuroinflammation. All of these hallmarks are considered potential AD therapeutic targets. All in all, these results underscore the pivotal role of DHA-richness nutritional strategies in the early intervention of AD and further suggest that increasing exposure to doses as high as 1.44 g/kg DHA does not add many additional benefits beyond those observed with DHA doses up to 0.88 g/kg.

Taken together, our data show the beneficial protection of DHA-enriched diets on neuronal count and brain health in a traditional mouse model for AD research, validating this hypothesis raised by this study. These promising findings open new powerful tracking avenues for further studies focused on the beneficial effects of DHA derived from sustainable and underexploited *Schizochytrium* sp. microalga in the prevention of AD.

As in non-clinical studies, this study bears a weakness. A limitation of this study is the absence of wild-type (WT) control groups for each dietary condition, which restricts the ability to assess the severity of cognitive deficits in 5×FAD mice and the specific impact of each diet. Without WT comparisons, it is unclear whether the diets improve cognition independently or primarily act to slow Alzheimer’s-related decline. Although relevant studies in WT mice supplemented with similar lipids provide context, the translational relevance remains limited. One should remember that human AD follows a slow, multi-decade progression, while murine models, due to genetic modifications, develop AD-like symptoms rapidly. This major difference largely affects translational accuracy [67,94] regarding AD onset and progression. Moreover, about genetic influence, murine models rely on specific mutations for AD onset, lacking the genetic complexity seen in humans, such as the influence of the APOE4 allele [110,111,112]. This limits their ability to mimic the multifactorial nature of human AD [113,114]. Hence, clinical studies are being planned in order to confirm the translation of the results accomplished in this non-clinical study.

## Figures and Tables

**Table 2:** Effect of DHA-enriched diets on plasma biochemistry profile of 5×FAD mice.

	Control	LSO	FO	Schizo	DHASCO	SEM	*p*-Value
Glucose (mg/dL)	107 ^b^	113 ^b^	113 ^b^	110 ^b^	131 ^a^	2.02	<0.001
Insulin (mU/L)	<0.4	<0.4	<0.4	<0.4	<0.4	-	-
Urea (mg/dL)	51.0 ^ab^	49.9 ^ab^	53.9 ^a^	46.9 ^b^	52.5 ^ab^	1.56	0.035
Creatinine (mg/dL)	0.080 ^a^	0.076 ^ab^	0.078 ^ab^	0.076 ^ab^	0.065 ^b^	0.004	0.044
Cholesterol (mg/dL)	84.9 ^a^	70.3 ^c^	61.1 ^d^	75.4 ^bc^	81.5 ^ab^	2.16	<0.001
LDL-CHR (mg/dL)	8.50 ^bc^	9.75 ^ab^	11.1 ^a^	7.50 ^c^	7.50 ^c^	0.487	<0.001
HDL-CHR (mg/dL)	73.0 ^a^	53.8 ^bc^	44.9 ^c^	66.0 ^a^	63.4 ^ab^	2.53	<0.001
VLDL-CHR (mg/dL)	13.6 ^ab^	14.1 ^a^	12.5 ^b^	12.9 ^ab^	13.6 ^ab^	0.325	0.013
Total lipids (mg/dL)	388 ^a^	361 ^c^	335 ^d^	366 ^bc^	381 ^ab^	4.09	<0.001
TAG (mg/dL)	68.1 ^ab^	70.5 ^a^	62.5 ^b^	64.8 ^ab^	67.8 ^ab^	1.63	0.013
ALT (U/L)	30.9 ^a^	22.4 ^b^	26.1 ^ab^	26.0 ^ab^	25.3 ^b^	1.36	0.003
AST (U/L)	156 ^c^	137 ^c^	283 ^a^	163 ^c^	226 ^b^	10.9	<0.001
GGT (U/L)	2.00	1.75	1.63	1.88	2.38	0.317	0.519
Total protein (g/dL)	8.37	8.54	8.49	8.55	8.45	0.077	0.474
C-reactive protein (mg/dL)	0.008 ^b^	0.018 ^a^	0.004 ^b^	0.007 ^b^	0.003 ^b^	0.002	<0.001
IGF-1 (µg/L)	<7.00	<7.00	<7.00	<7.00	<7.00	-	-
IL-6 (ng/L)	<1.5	<1.5	<1.5	<1.5	<1.5	-	-

Control: AIN-93M diet; LSO: linseed oil diet; FO: cod liver oil diet; Schizo: *Schizochytrium* microalga oil diet; DHASCO: commercial DHASCO oil diet; ALT: alanine aminotransferase (EC 2.6.1.2); AST: aspartate aminotransferase (EC 2.6.1.1); GGT: glutamyltransferase (EC 2.3.2.13); CHR: cholesterol; HDL: high-density lipoprotein; LDL: low-density lipoprotein; VLDL: very low-density lipoprotein; TAG: triacylglycerol; IGF-1: insulin growth factor 1; IL-6: interleukin-6. VLDL cholesterol = 1/5 [TAG], as described by Friedewald et al. [41]. Total lipids = 2 × [total cholesterol] + [TAG] + 150, as derived from the formula described by Covaci et al. [42]. Data are mean ± SEM (standard error of the mean). Different superscript letters within a row indicate a significant difference (*p* < 0.05).

**Table 3:** Effect of DHA-enriched diets on the main fatty acids determined in the liver of 5×FAD mice (results are expressed as % of total fatty acids).

	Control	LSO	FO	Schizo	DHASCO	SEM	*p*-Value
14:0	0.472 ^ab^	0.425 ^b^	0.526 ^ab^	0.670 ^a^	0.375 ^b^	0.060	0.014
16:0	21.2 ^c^	21.1 ^c^	23.6 ^b^	25.0 ^a^	25.5 ^a^	0.331	<0.001
16:1n-9	0.734 ^a^	0.735 ^a^	0.831 ^a^	0.586 ^ab^	0.446 ^b^	0.067	0.003
16:1n-7	3.98 ^a^	2.90 ^ab^	3.85 ^a^	3.55 ^a^	2.00 ^b^	0.305	<0.001
18:0	7.89	8.04	6.48	6.35	8.39	0.646	0.098
18:1n-9	22.9 ^a^	20.8 ^a^	22.7 ^a^	19.8 ^ab^	15.1 ^b^	1.31	0.001
18:1n-7	2.44 ^a^	1.78 ^b^	2.17 ^ab^	1.69 ^b^	0.996 ^c^	0.151	<0.001
18:2n-6	19.4	20.7	20.4	20.8	20.2	1.02	0.874
19:0	0.518 ^a^	0.350 ^abc^	0.402 ^ab^	0.322 ^bc^	0.215 ^c^	0.044	<0.001
18:3n-3	0.674 ^b^	3.12 ^a^	0.849 ^b^	0.935 ^b^	0.739 ^b^	0.211	<0.001
21:0	0.855 ^ab^	0.928 ^a^	0.835 ^ab^	0.510 ^b^	0.509 ^b^	0.087	0.002
20:4n-6	9.40 ^a^	6.70 ^b^	4.72 ^bc^	4.21 ^c^	5.67 ^bc^	0.602	<0.001
20:5n-3	0.215 ^b^	1.18 ^a^	1.32 ^a^	1.38 ^a^	1.23 ^a^	0.097	<0.001
22:5n-6	0.183 ^c^	0.005 ^d^	0.000 ^d^	0.486 ^b^	1.02 ^a^	0.043	<0.001
22:5n-3	0.340 ^c^	0.901 ^a^	0.745 ^ab^	0.630 ^b^	0.672 ^b^	0.048	<0.001
22:6n-3	6.01 ^c^	7.35 ^c^	7.37 ^c^	10.9 ^b^	14.8 ^a^	0.604	<0.001
SFA	31.5 ^b^	31.4 ^b^	32.4 ^b^	33.4 ^ab^	35.5 ^a^	0.675	<0.001
MUFA	30.6 ^a^	26.7 ^a^	30.4 ^a^	26.0 ^a^	18.8 ^b^	1.75	<0.001
PUFA	36.8 ^b^	40.7 ^ab^	35.9 ^b^	39.7 ^ab^	44.7 ^a^	1.44	0.001
n-3 PUFA	7.46 ^d^	13.1 ^b^	10.6 ^c^	14.0 ^b^	17.7 ^a^	0.582	<0.001
n-6 PUFA	29.3	27.5	25.2	25.6	27.0	1.04	0.066
n-3/n-6	0.255 ^d^	0.477 ^bc^	0.421 ^c^	0.553 ^b^	0.658 ^a^	0.021	<0.001

Control: AIN-93M diet; LSO: linseed oil diet; FO: cod liver oil diet; Schizo: *Schizochytrium* microalga oil diet; DHASCO: commercial DHASCO oil diet. SFA: saturated fatty acid; MUFA: monounsaturated fatty acid; and PUFA: polyunsaturated fatty acid. The n-3/n-6: ratio obtained through the division of n-3 PUFA by n-6 PUFA. Data are mean ± SEM (standard error of the mean). Different superscript letters within a row indicate a significant difference (*p* < 0.05).

**Table 4:** Effect of DHA-enriched diets on the main fatty acids determined in the brain of 5×FAD mice (results are expressed as % of total fatty acids).

	Control	LSO	FO	Schizo	DHASCO	SEM	*p*-Value
16:0	19.3	19.0	19.2	19.0	19.4	0.266	0.732
16:1n-9	0.177 ^ab^	0.179 ^a^	0.179 ^a^	0.156 ^bc^	0.153 ^c^	0.006	0.001
16:1n-7	0.615	0.653	0.668	0.653	0.630	0.018	0.271
16:3n-4	0.357	0.379	0.316	0.358	0.336	0.021	0.266
16:3n-3	3.22	3.17	3.23	3.27	3.04	0.112	0.628
16:4n-3	1.21	1.25	1.22	1.24	1.17	0.048	0.785
18:0	20.0	19.9	19.7	19.7	19.9	0.148	0.477
18:1n-9	14.9 ^b^	15.6 ^ab^	15.8 ^ab^	16.0 ^a^	15.7 ^ab^	0.240	0.029
18:1n-7	3.42 ^a^	3.32 ^abc^	3.37 ^ab^	3.23 ^bc^	3.19 ^c^	0.037	<0.001
18:2n-6	0.524 ^b^	0.655 ^a^	0.633 ^a^	0.643 ^a^	0.628 ^ab^	0.026	0.008
20:1n-9	1.37	1.43	1.57	1.46	1.50	0.092	0.639
20:4n-6	9.35 ^a^	8.71 ^b^	8.18 ^b^	7.42 ^c^	7.31 ^c^	0.141	<0.001
20:5n-3	0.000 ^c^	0.075 ^b^	0.104 ^b^	0.159 ^a^	0.174 ^a^	0.008	<0.001
22:4n-6	2.47 ^a^	2.23 ^b^	1.91 ^c^	1.57 ^d^	1.49 ^d^	0.041	<0.001
22:5n-6	0.221 ^c^	0.000 ^d^	0.001 ^d^	0.728 ^b^	0.997 ^a^	0.033	<0.001
22:5n-3	0.102 ^b^	0.294 ^a^	0.298 ^a^	0.295 ^a^	0.313 ^a^	0.015	<0.001
22:6n-3	15.9 ^b^	15.9 ^b^	16.2 ^ab^	16.8 ^a^	16.9 ^a^	0.201	<0.001
SFA	40.9	40.7	40.8	40.4	41.2	0.341	0.637
MUFA	21.1	21.8	22.3	22.2	21.9	0.359	0.182
PUFA	33.4 ^a^	32.8 ^ab^	32.3 ^b^	32.6 ^ab^	32.5 ^ab^	0.238	0.014
n-3 PUFA	20.4 ^b^	20.7 ^b^	21.1 ^ab^	21.7 ^a^	21.6 ^a^	0.178	<0.001
n-6 PUFA	12.7 ^a^	11.7 ^b^	10.8 ^c^	10.5 ^c^	10.5 ^c^	0.154	<0.001
n-3/n-6	1.61 ^d^	1.77 ^c^	1.95 ^b^	2.08 ^a^	2.06 ^a^	0.029	<0.001

Control: AIN-93M diet; LSO: linseed oil diet; FO: cod liver oil diet; Schizo: *Schizochytrium* microalga oil diet; and DHASCO: commercial DHASCO oil diet. SFA: saturated fatty acid; MUFA: monounsaturated fatty acid; and PUFA: polyunsaturated fatty acid. n-3/n-6: ratio obtained through the division of n-3 PUFA by n-6 PUFA. Data are mean ± SEM (standard error of the mean). Different superscript letters within a row indicate a significant difference (*p* < 0.05).

## Data Availability

Data generated within this study are included in the published article. All datasets produced during the experiment are available upon request from the corresponding author.
